# Electrospun polycaprolactone membranes with Zn-doped bioglass for nasal tissues treatment

**DOI:** 10.1007/s10856-019-6280-4

**Published:** 2019-06-26

**Authors:** Izabella Rajzer, Michał Dziadek, Anna Kurowska, Katarzyna Cholewa-Kowalska, Magdalena Ziąbka, Elżbieta Menaszek, Timothy E. L. Douglas

**Affiliations:** 1 Department of Mechanical Engineering Fundamentals, Division of Materials Engineering, ATH University of Bielsko-Biala, Willowa 2 street, 43-309 Bielsko-Biała, Poland; 2 Department of Glass Technology and Amorphous Coatings, Faculty of Materials Science and Ceramics, AGH University of Science and Technology, Krakow, Poland; 3 Department of Ceramics and Refractories, Faculty of Materials Science and Ceramics, AGH University of Science and Technology, Krakow, Poland; 4 Department of Cytobiology, UJ Jagiellonian University, Collegium Medicum, Krakow, Poland; 5 Engineering Department, Lancaster University, Lancaster, United Kingdom; 6 Materials Science Institute (MSI), Lancaster University, Lancaster, United Kingdom

## Abstract

In this work, composite membranes were investigated as future components of a layered implant for the reconstruction of nasal septum. Incorporation of zinc ions into nasal implants could potentially provide antibacterial properties to decrease or eliminate bacterial infections and subsequent surgical complications. Two types of membranes were prepared using an electrospinning method: PCL with bioglass and PCL with bioglass doped with Zn. The aim of this work was to investigate the influence of bioglass addition on the morphology, fiber diameter and composition of the membranes. The apatite-forming ability was examined in Simulated Body Fluid (SBF). The cytotoxicity of the membranes, ALP activity and in vitro mineralization were evaluated in cell culture. The mineralization and ALP activity was higher for polycaprolactone membranes modified with Zn doped bioglass than compared to pure PCL membranes or control material. The results proved that the presence of Zn^2+^ in the electrospun membranes = influence the osteogenic differentiation of cells.

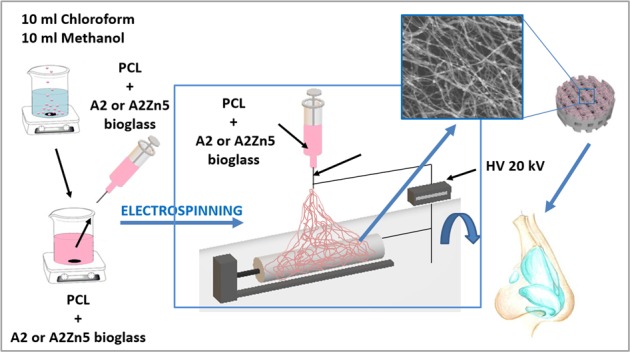

## Introduction

Injuries of nasal bone and cartilage are important issues not only for the patients but also for laryngologists and plastic surgeons [1]. Moreover selecting and tailoring the appropriate material to reconstruct the nose functionally and esthetically is crucial to ensure successful surgery [2]. Autologous cartilage is the most biologically acceptable augmentation material in rhinoplasty [3]. However, its applicability is limited by the finite availability and donor postoperative pain. Considering these disadvantages, the use of alloplasts in rhinoplastic or septoplastic procedures seems to be extremely attractive because of the lack of donor site morbidity, unlimited availability, ease of contouring, and relative simplicity of insertion. The most common materials used as nasal alloplasts are Gore-Tex® (based on polytetrafluoroethylene, PTFE), Lactosorb® (made of poly-L-lactic acid and polyglycolic acid), silicone, and Med-Por® (based on porous, high-density polyethylene HDPE) [4–6]. During material implantation, bacteria can easily adhere to and colonize the biomaterial surfaces, ultimately leading to serious implant infection. Complications from infections and aggressive foreign body reactions sometimes lead to extrusion and damage of overlying tissue [7].

One of the materials recently used in clinical study for rhinoplasty is polycaprolactone (PCL) [8]. PCL is a biodegradable and biocompatible material with good flexibility and mechanical properties [9]. PCL exhibits more prolonged mechanical strength than other bioresorbable polymeric materials, and the degradation period of PCL graft is sufficient to allow integration with replacement by viable host tissue [8]. PCL is a safe material for implants in nasal reconstruction, showing good stability via incorporation into the host tissue and maintenance of the immune response [2, 10]. Another material which was successfully used in nasal reconstruction surgery, showing good results in the reconstruction of perforation of the nasal septum, was bioglass (BG) [11]. BG is a type of bioactive ceramic material generally composed by SiO_2_–CaO–P_2_O_5_ and Na_2_O and it can mainly be synthesized through two approaches: melting or sol–gel [12]. The major advantage of BG is its excellent bioactivity. Therefore, materials containing BG are widely used for bone tissue engineering, and have been demonstrated to be beneficial to osteogenesis in vitro and in vivo [13]. Moreover a variety of studies have recently focused on enhancing the antibacterial performance of bioactive glasses by doping them with antibacterial metallic ions (copper, cerium, zinc, silver) [14].

Incorporation of zinc ions into nasal implants could potentially provide antibacterial properties to decrease or eliminate bacterial infections and the subsequent complications in the surgery. Moreover, Zn-based biomaterials have promising applications in tissue regeneration because of their biocompatibility, osteogenesis and anticancer properties [15, 16]. The Zn ion is well known for its roles in bone growth through promoting osteoblast and chondrocyte differentiation, while inhibiting osteoclastic bone resorption [15, 17]. A wide variety of methods have been used to produce materials for osteochondral defect treatment in the forms of porous sponges, woven or non-woven meshes and hydrogels [18–20]. Electrospinning is a simple, cost-effective and versatile method to prepare materials that mimic the native architecture of tissues. Electrospun scaffolds have attracted considerable interest in tissue engineering [21]. Moreover the possibility to modify the polymer solution used for electrospinning with bioactive substances or drugs make it an attractive method for nasal implant development.

In this work, composite membranes were investigated as a future component of a layered implant for the reconstruction of nasal septum. The nasal septum is a bony partition and cartilage within the nasal cavity. Because the cartilage of the nasal septum connects to the nasal bone, our ultimate intention is to develop a layered scaffold composed of 3D printed bottom layer (for the cartilaginous part of implant) and an electrospun upper layer (which will have contact with bone). This work is focused on the production and characterization of electrospun membranes for the upper layer of the nasal scaffold. Two types of membranes were prepared using an electrospinning method: PCL with bioglass and PCL with bioglass doped with Zn. The aim of this study was to investigate the influence of bioglass addition on the morphology, fiber diameter and composition of the membranes. The apatite-forming ability was examined in Simulated Body Fluid (SBF). The cytotoxicity of the membranes, ALP activity and in vitro mineralization were evaluated in cell culture using normal human osteoblasts.

## Materials and methods

### Materials

Bioactive glass (BG) A2 of the following composition (mol%) 40SiO_2_–54CaO–6P_2_O_5_ was produced using the sol–gel method [22]. Two glass particle sizes were obtained: <45 μm by grinding and sieving and around 1.0 μm by milling in an attritor with ZrO_2_ balls in isopropyl alcohol medium.

The zinc-doped bioactive glass A2Zn5 (mol%): 49CaO–5ZnO–6P_2_O_5_–40SiO_2_ was synthetized using a sol–gel method [23]. Tetraethyl orthosilicate (TEOS, Si(OC_2_H_5_)_4_), triethyl phosphate (TEP, OP(OC_2_H_5_)_3_) (Sigma-Aldrich, USA), calcium nitrate tetrahydrate (Ca(NO_3_)_2_·4H_2_O), and zinc nitrate hexahydrate (Zn(NO_3_)_2_·6H_2_O) (POCH, Poland) were used as the starting materials. 1 M HCl solution (POCH, Poland) was applied as a catalyst of the hydrolysis and polycondensation reactions. The resulting solutions were left at the ambient conditions until the gels were formed. After drying at 80 °C, gels were heated up to 700 °C. The gel-derived materials were then milled in an attritor with ZrO_2_ grinding balls in isopropyl alcohol medium. Particle size distribution in powder aqueous suspension was analyzed using laser diffraction method (Mastersizer 2000, Malvern, UK). The resulting particle size was 1.5 µm (d_50_).

Polycaprolactone (PCL) with an average molecular weight of 80 kDa was purchased from Sigma-Aldrich. Chloroform and methanol 1:1 (POCH, Poland) were used as solvents. To prepare the spinning solutions, 1 g of PCL was dissolved in 10 mL of chloroform/methanol (1:1) mixture. A2 and A2Zn5 BG powders were added into the polycaprolactone solutions. In order to investigate the effect of different particle size of BG on the microstructure of PCL-BG membranes, two different solutions with different BG particle sizes (~1.0 μm and <45 μm) were prepared. We have also investigated the effects of BG concentration and solution flow rate, therefore two different BG concentrations (1) 2 wt% and (2) 4 wt% and three different solution flow rates during electrospinning were applied: (1) 5 mL/h (2) 3 mL/h and (3) 1 mL/h. Stable dispersion of bioglass powder was achieved by sonicating the slurry.

### Scaffold fabrication by electrospinning

Membranes were developed using an electrospinning method (TIC 1092012 machine, ATH, Bielsko-Biała). For membranes fabrication, samples of prepared solutions were placed in a syringe (10 mL) topped with a needle whose diameter was 0.22 mm and then connected to 25 kV voltage. The distance between the needle and the collector was 20 cm. The rotary drum was wrapped in silicone coated paper. Two membranes with different A2 particle sizes (1.5 µm, 45 µm) were prepared. Moreover, three different solution flow rates were applied 5, 3, 1 mL/h. Finally, to prepare PCL-A2 and PCL-A2Zn5 membranes, A2 and A2Zn5 BG with particle sizes of 1.0 and 1.5 µm, 4 wt% powder concentration in the solution and 3 mL/h flow rate were chosen.

### Scaffold characterization

A Nova NanoSEM 200 scanning electron microscope (SEM; FEI, Eindhoven, The Netherlands) coupled with a GenesisXM X-ray microanalysis system (EDAX, Tilburg, The Netherlands) equipped with a Sapphire Si(Li) energy dispersive X-ray (EDX) detector was used to perform the examination of the microstructure of the produced membranes. The microstructures of scaffolds were evaluated after coating with a carbon layer. The average fiber diameter was calculated based on SEM images. Approximately 100 fibers were analyzed using the Image J software.

Infrared spectroscopy was performed with a Bruker VERTEX 70 V spectrometer (ATR technique with the use of a platinum single-crystal diamond for membranes. For A2 and A2Zn5 powders, KBr pellets were fabricated, by dispersing the sample in KBr. All spectra were collected in the range of 4000–550 cm^−1^, 128 scans were accumulated at 4 cm^−1^ resolution.

### Bioactivity assessment

The bioactivity of the obtained membranes was evaluated by examining the formation of apatite layer on their surfaces in SBF. Simulated body fluid solution (1.5 × SBF, pH 7.4) was prepared according to Kokubo et al. [24]. Three membrane samples of PCL, PCL-A2 and PCL-A2Zn5 were immersed in 1.5 × SBF solution and stored at the temperature of 37 °C for 7 days. The solution was replaced every 2.5 days and an apatite layer was allowed to nucleate and grow on the surface of the samples. After 7 days the samples were removed from SBF and dried at room temperature. In order to investigate the effect of bioglass content on the properties of membranes, SEM/EDX evaluation and FTIR analysis were performed.

### Cell culture study

In this work, the Normal Human Osteoblasts cells (NHOst, Lonza, USA) were used to assess the membranes’ cytocompatibility. Prior to cell seeding, cells were expanded in osteoblast growth medium OGM BulletKit (Lonza, USA) containing 10% FBS, 0.1% ascorbic acid and 0.1% GA-1000 (Gentamicin Sulfate and Amphotericin-B) at 37 °C in a humidified incubator with 5% CO_2_. The cultured medium was renewed every 3 days.

The cell culture experiment was carried out with three types of electrospun membranes: (1) PCL, (2) PCL-A2, (3) PCL-AnZn5. The selected materials were cut into disks (round samples matching the size of wells of 48-well culture plate), sterilized by soaking in 70% ethanol for 30 min and by exposure to UV light for 30 min (each side) and then washed with sterile phosphate buffered saline (PBS, HyClone, USA). The membranes were placed at the bottom of 48-well culture plates and seeded with cells at a cell of concentration 1.5 × 10^4^ cells/mL/well. An empty polystyrene well served as a positive control (TCPS). NHOst cells were cultured on the materials for 7, 14, and 21 days in complete osteoblast growth medium OGM supplemented with differentiation kit SingleQuots (Lonza, USA), containing hydrocortisone-21-hemisuccinate and β-glycerophosphate.

#### Cell morphology observation

Detailed morphological examination of the cells which adhered to the investigated materials was performed using SEM method (Nova NanoSEM 200 FEI Europe Company) and fluorescence microscope (Olympus CX41, Japan).

The SEM measurements and observations were conducted in low vacuum conditions, with LVD and Helix detector at an accelerated voltage of 10–18 kV. After 21 days of cell culture, the materials were washed with PBS. The cells were fixed with 3% glutaraldehyde solution in sodium cacodylate buffer at pH 7.4 (POCh, Gliwice, Poland) for 0.5 h and then dehydrated in a series of ethanol solution (70%, 80%, 90%, 96%, 100%), dried in air and evaluated using SEM.

Cell morphologies were evaluated after 14 days of cell culture using fluorescence microscopy. The cells were stained for 1 min with 0.01% acridine orange (AO) solution (Sigma-Aldrich, USA), rinsed with PBS and photographed.

#### Cell proliferation and membranes cytotoxicity

Cell proliferation/cytotoxicity tests (ToxiLight BioAssay Kit and ToxiLight, Lonza USA) were conducted at day 7 and 14. The kit was used to quantify adenylate kinase (AK) in both lysate (representing intact adherent cells) and supernatant (representing damaged cells). The results were expressed as mean ± standard deviation (SD) from 8 samples for each experimental group.

#### Alkaline phosphatase activity

Alkaline phosphatase (ALP) activity was evaluated using 4-Methylumbelliferyl phosphate (MUP) as a substrate. The hydrolysis of MUP was determined by fluorescence detection on POLARStar Omega microplate reader (BMG Labtech, Germany) with setting for excitation at 360 and emission at 440 nm on the 7th and 14th day of culture.

#### In vitro mineralization in cell culture

Cell culture mineralization was evaluated by the OsteoImageTM Mineralization Assay (Lonza, USA) according to the manufacturer’s instructions. OteoImage assay is based on the fluorescent staining of extracellular mineral content deposited by cells, specifically hydroxyapatite. The test was performed after 14 and 21 days of the cell culture. Mineralization-stained images were obtained at 490/520 nm excitation/emission wavelengths using POLARstar Omega microplate reader. The results were expressed as mean ± standard deviation (SD) from 4 samples for each experimental group.

#### Statistical analysis

The analysis of variance (ANOVA) was used to determine the differences among the evaluated series of samples. Then Duncan Post Hoc tests, which were performed with Statistica 10 (StatSoft^®^, USA) software were applied. The results were considered statistically significant when *p* < 0.05.

## Results

### Morphological characterization of electrospun membranes

**Fig. 1:**
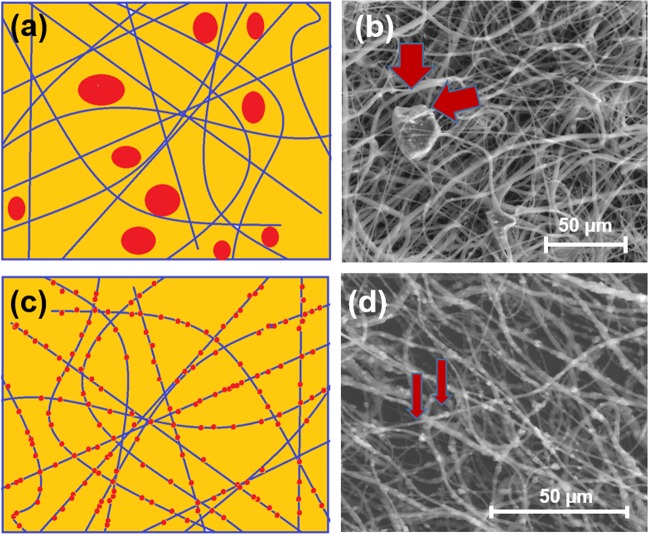
Scheme and SEM images of bioglass particle distribution within fibrous scaffold. **a**, **b** Size of the particles lower than 45 µm, particles between the fibers. **c**, **d** Size of the particles around 1 µm, particles are incorporated into the fibers

PCL-BG membranes were produced by electrospinning process using two different sizes of BG particles: (1) <45 µm and (2) around 1.0 µm. The morphology of the obtained electrospun membranes was investigated by SEM (Fig. 1). In the case of bigger particle size (<45 µm) it was observed that the majority of the particles were located between the fibers (Fig. 1a, b). Rough fiber surfaces were obtained in the case of PCL-BG membranes produced with addition of the small size of A2 bioglass particles. A2 particles were located inside the fibers and on the surface of fibers (Fig. 1c, d). On the basis of obtained result for subsequent electrospinning process, the BG powder with the smaller particle size was chosen.

**Fig. 2:**
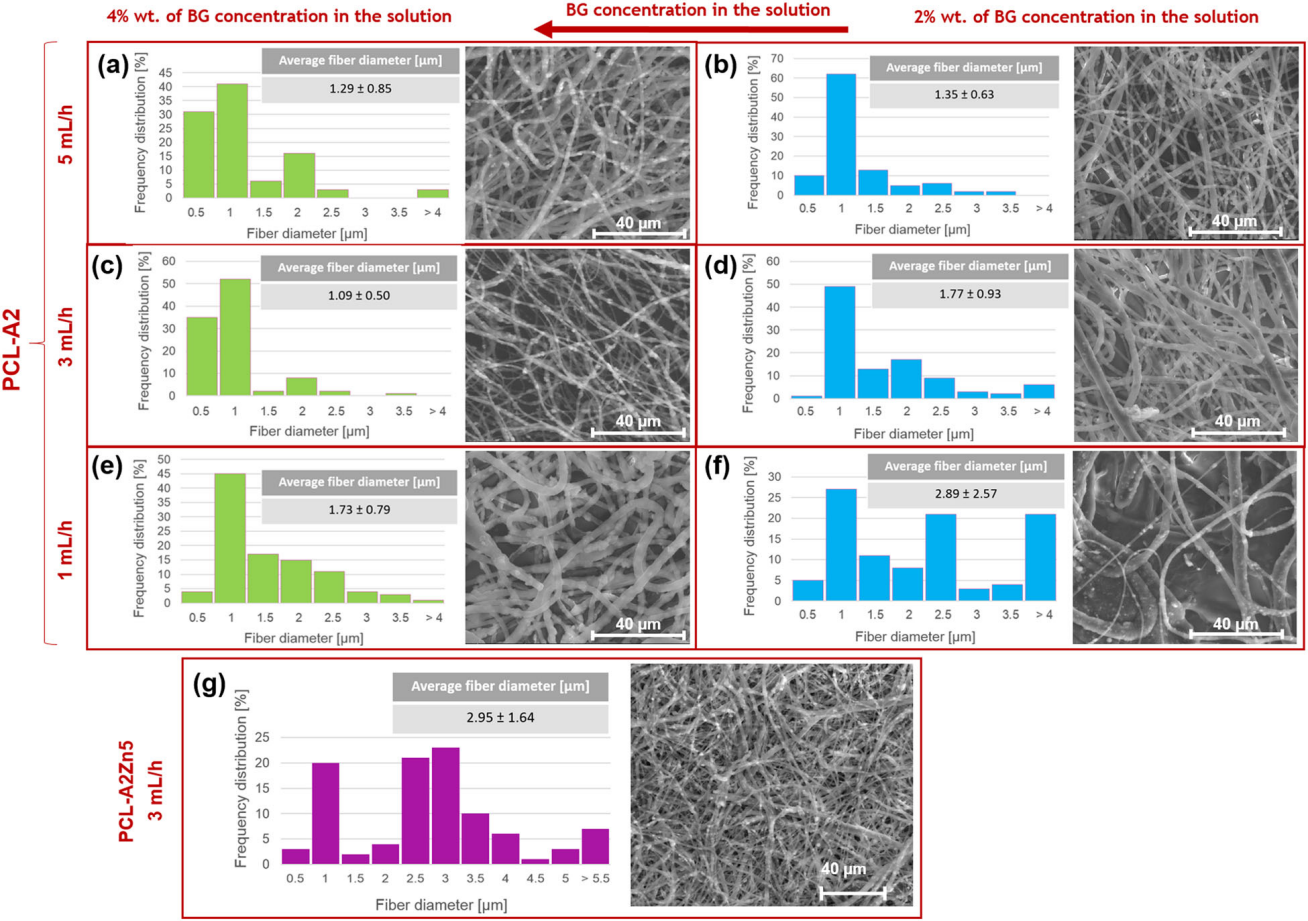
SEM images of the fibrous PCL-A2 membranes together with fiber diameter distribution. **a**, **c**, **e** 4% wt. of A2 bioglass concentration. **b**, **d**, **f** 2% wt. of A2 bioglass addition. Samples prepared at different flow ratio respectively 5, 3, 1 mL/h, PCL-A2Zn5 membrane prepared at 3 mL/h (**g**)

Figure 2 shows the SEM images and the fiber diameter distribution of PCL-A2 membranes produced at three different flow rates (1,3,5 mL/h) and different bioglass concentration (2 and 4 wt%). As a result of electrospinning method we have obtained membranes with randomly oriented fibers and high porosity due to the high ratio of surface area to volume. PCL-A2 membranes are formed by the long fibers with rough surfaces. BG particles could be observed on the surface of fibers. From the SEM images, it is clear that A2 bioglass particles were successfully incorporated into all of the obtained membranes. The dispersion of the bioactive ceramic particles was heterogeneous and some agglomeration of particles was also noticed. Fiber diameter range from 500 nm to over 4 µm. The average diameter of PCL-A2 membrane fibers depends on the electrospinning flow rate and on the concentration of BG (Fig. 2). The smallest average diameter of the composite fibers (1.09 ± 0.5 µm) was obtained for the concentration of 4 wt% BG and a flow rate of 3 mL/h, and this sample was selected for further study.

In the case of Zn-doped PCL-A2Zn5 membrane particles of BG were slightly bigger (1.5 µm) resulting in bigger average fiber diameter (2.95 µm ± 1.64). The quite large standard deviation of the average fiber diameter results from the tendency of the powder to agglomerate. Although particles are agglomerated, they are well distributed in the fibrous membrane (Fig. 2g).

**Fig. 3:**
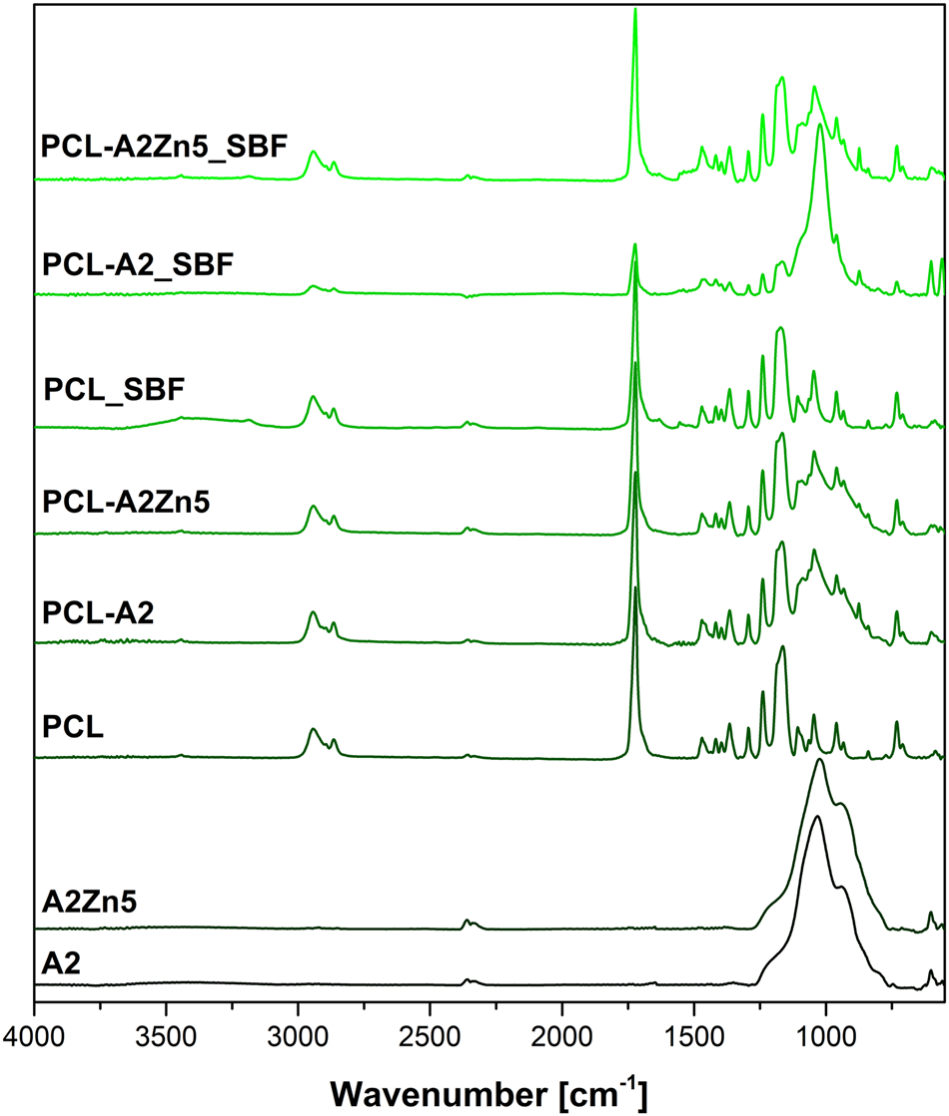
FTIR spectra of electrospun membranes: PCL, PCL-A2, PCL-A2Zn5 before and after incubation in SBF and pure A2 and A2Zn5 powder

The surface chemistry was characterized by FTIR analysis. As shown in Fig. 3, in the spectrum of bioglass A2 and A2Zn, the band located at 1020 cm^−1^ correspond to stretching vibration of [PO_4_] as well as [SiO_4_] units. The minor band at 930 cm^−1^ corresponds to Si–O stretching of non-bridging oxygen atoms in SiO_4_ tetrahedra. Additionally small peaks at 600 cm^−1^ and 875 cm^−1^ reflect bending vibration bands of O–P–O and CO_3_^2−^ bending mode, respectively.

FTIR studies confirmed that A2 and A2Zn5 BG particles were successfully incorporated into the fibrous PCL membrane. In the spectra of PCL-A2 and PCL-A2Zn5 samples, the characteristic peaks of polycaprolactone are observed at 2949 (CH_2_ asymmetric, stretching) and 2865 cm^−1^ (CH_2_ symmetric, stretching), 1727 cm^−1^ (C = O stretching), 1293 cm^−1^ (C–O, C–C stretching), 1240 cm^−1^ (C–O–C asymmetric, stretching), 1175 cm^−1^ (symmetric stretching, 1157 cm^−1^ (C–O, C–C stretching). Moreover, the characteristic bands corresponding to the presence of BG in the sample, which do not appear in the case of pure polycaprolactone membrane, can be identified in both modified PCL-A2 and PCL-A2Zn5 samples.

### Bioactivity assessment in SBF

FTIR spectra were recorded (Fig. 3) in order to investigate the effect of BG content on the ability to promote formation of an apatite layer in SBF. After 1 week of soaking in SBF, FTIR spectrum of electrospun PCL-A2 membranes exhibit typical bands attributed to apatite. In detail, peaks at 560 and 600 cm^−1^ ascribable to phosphate group vibrations (O–P–O bending mode), 1014 cm^−1^ (P–O stretching mode), 875 cm^−1^ (CO_3_^2−^ bending mode) confirm the formation of apatite layer. After 7 days of immersion in SBF much more apatite forms on the surface of PCL-A2 membrane than on pure PCL or Zn-doped PCL-A2Zn5 membranes.

**Fig. 4:**
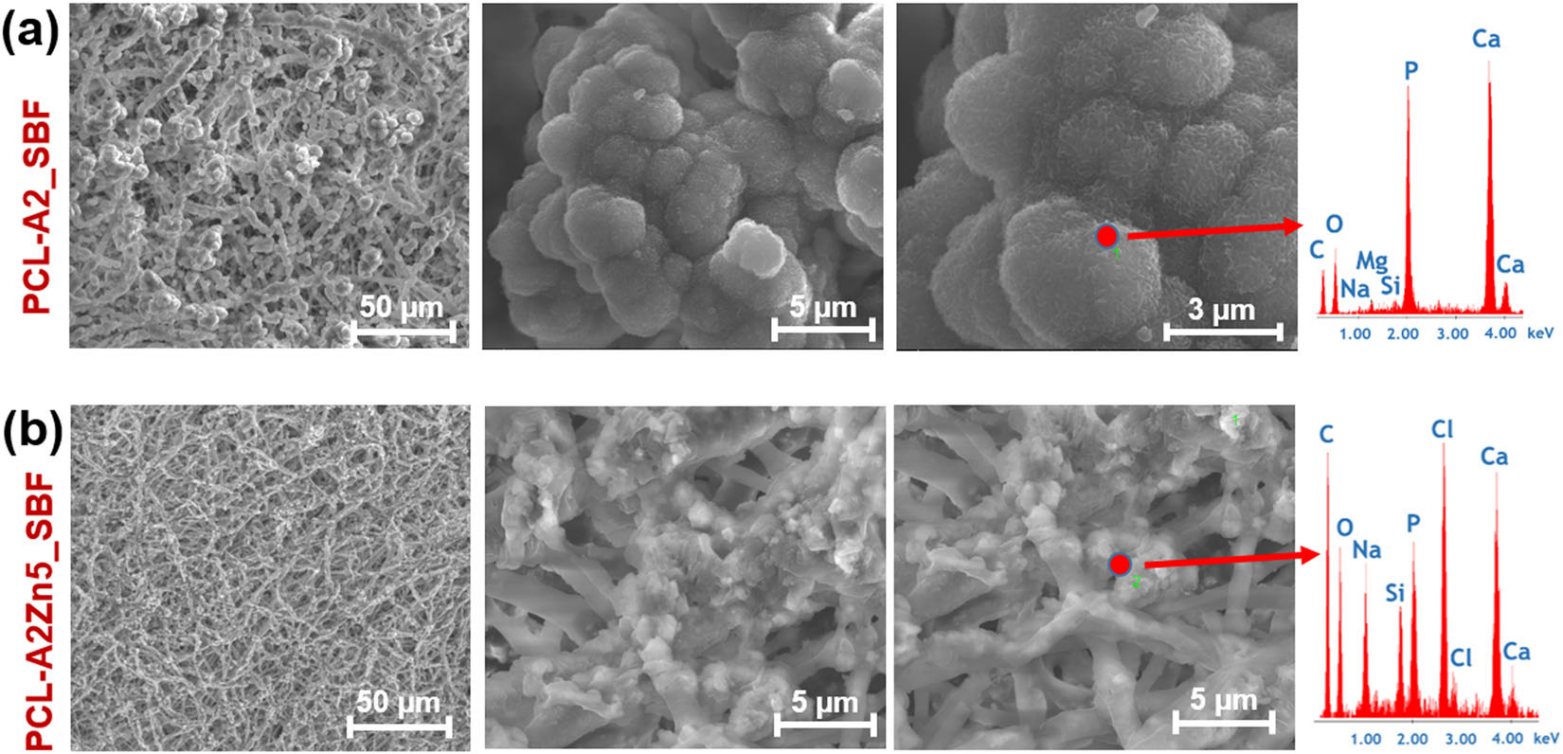
Microstructure of PCL-A2 and PCL-A2Zn5 electrospun scaffolds after 7 days of incubation in SBF

The presence of an apatite layer on the PCL_A2 membranes’ surface after 7 days of incubation in SBF was indicated by FTIR results (Fig. 3) and as well by inspection of SEM images (Fig. 4). SEM images confirmed the formation of a spherical cauliflower- like apatite on the surface of PCL-A2 membrane during the bioactivity study (Fig. 4a). According to the EDX results, the deposited minerals consist mainly of phosphorous (P) and calcium (Ca), proving the formation of an apatite layer on the surface of fibrous membranes.

In the case of Zn doped bioglass the formation of apatite layer after 7 days of sample incubation was much slower than in the case of unmodified samples and not so pronounced (Fig. 4b). Results of the EDX analysis shows that the deposited minerals, which can be observed on the membrane surface, are composed mainly of sodium (Na), chlorine (Cl), and small amounts of P and Ca. This indicates that mainly salt was formed on the surface of Zn-doped membranes.

Three types of membranes, namely (1) PCL-A2Zn5, (2) PCL-A2 and (3) Pure PCL were chosen for cell culture study.

### Cell culture

**Fig. 5:**
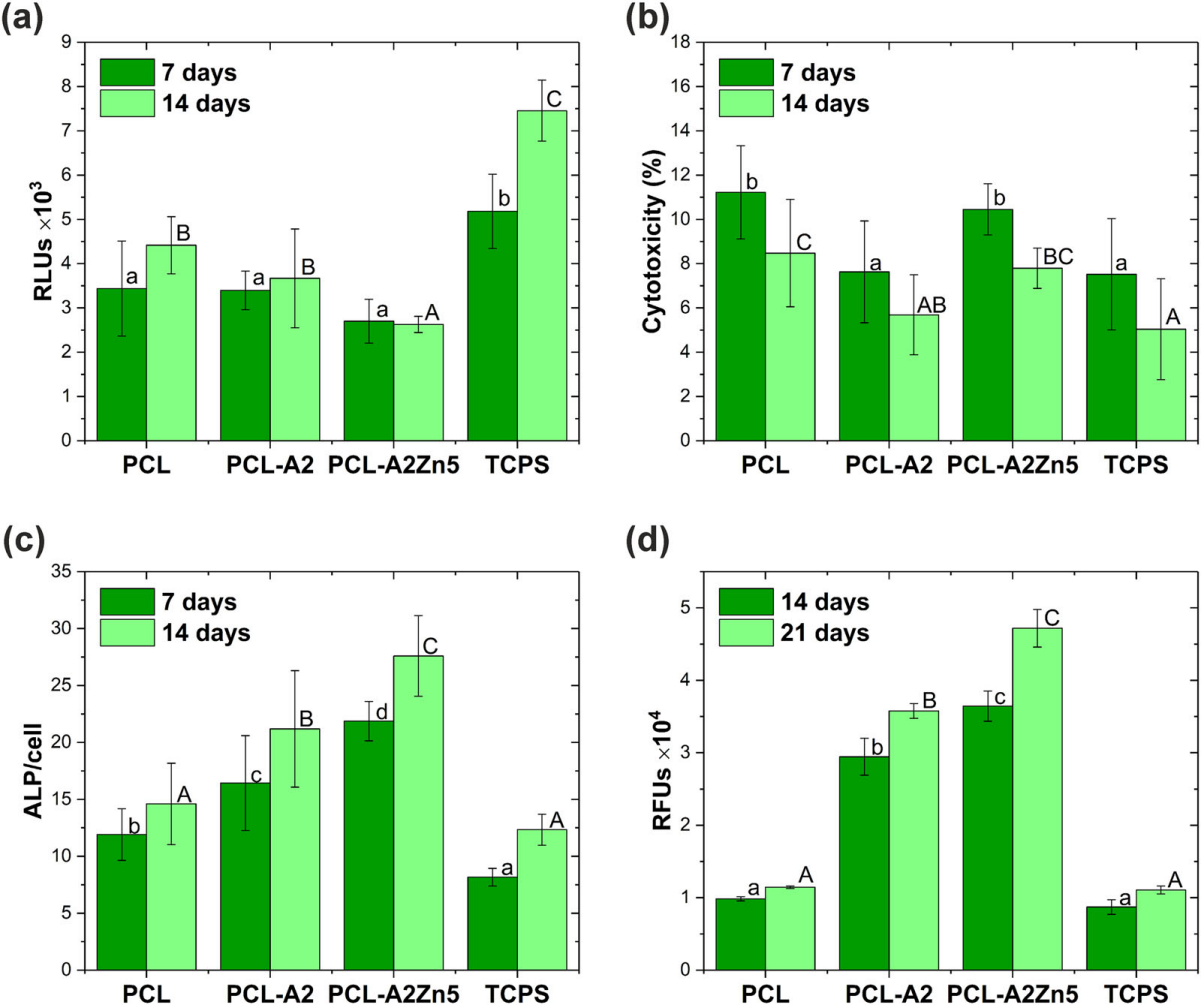
Number of intact adherent NHOst cells (**a**), Cytotoxicity (**b**), Alkaline phosphatase activity test (**c**), Mineralization progress. RFUs—relative fluorescence units (**d**). Results are expressed as mean ± SD. Statistically significant differences (*p* < 0.05) between each materials and TCPS after different cell culture periods are indicated by uppercase and lowercase letters, respectively

The number of cells attached per sample was measured using cell proliferation assays. Higher cell proliferation was observed in the case of control TCPS (Fig. 5a). The lower cell numbers on fibrous membranes is due to their highly porous structures. The lowest number of cells was observed in the presence of the PCL-A2Zn5 sample; the cells divided more slowly, probably due to induction of their differentiation. Cytotoxicity of the electrospun materials, estimated on the basis of the measurement of adenylate kinase (AK) released from the damaged cells on the 7th and 14th day of the culture is presented in Fig. 5b. The values of the cytotoxicity test were much lower after 14 days of cell culture.

ALP is an early indicator of immature osteoblasts’ activity and plays an important role in the bone matrix mineralization process. The results of ALP activity assay are given in Fig. 5c. ALP production increases on all samples from day 7 to 14. On day 14, the ALP activity of NHOst cells cultured on BG modified membranes: PCL-A2Zn5 and PCL-A2 was significantly higher than the ALP activity of the cells cultured on control TCPS. The presence of BG doped with Zn ions within the samples favors ALP expression and causes stimulation of cells in comparison to pure PCL membranes.

The ability of NHOst cells to deposit minerals is an indicator of mature osteoblasts and osteogenic efficiency. For materials modified with a BG, a statistically significant increase in the OsteoImage (OI) test value was observed compared to the control and pure PCL, indicating cells’ differentiation (Fig. 5d).

**Fig. 6:**
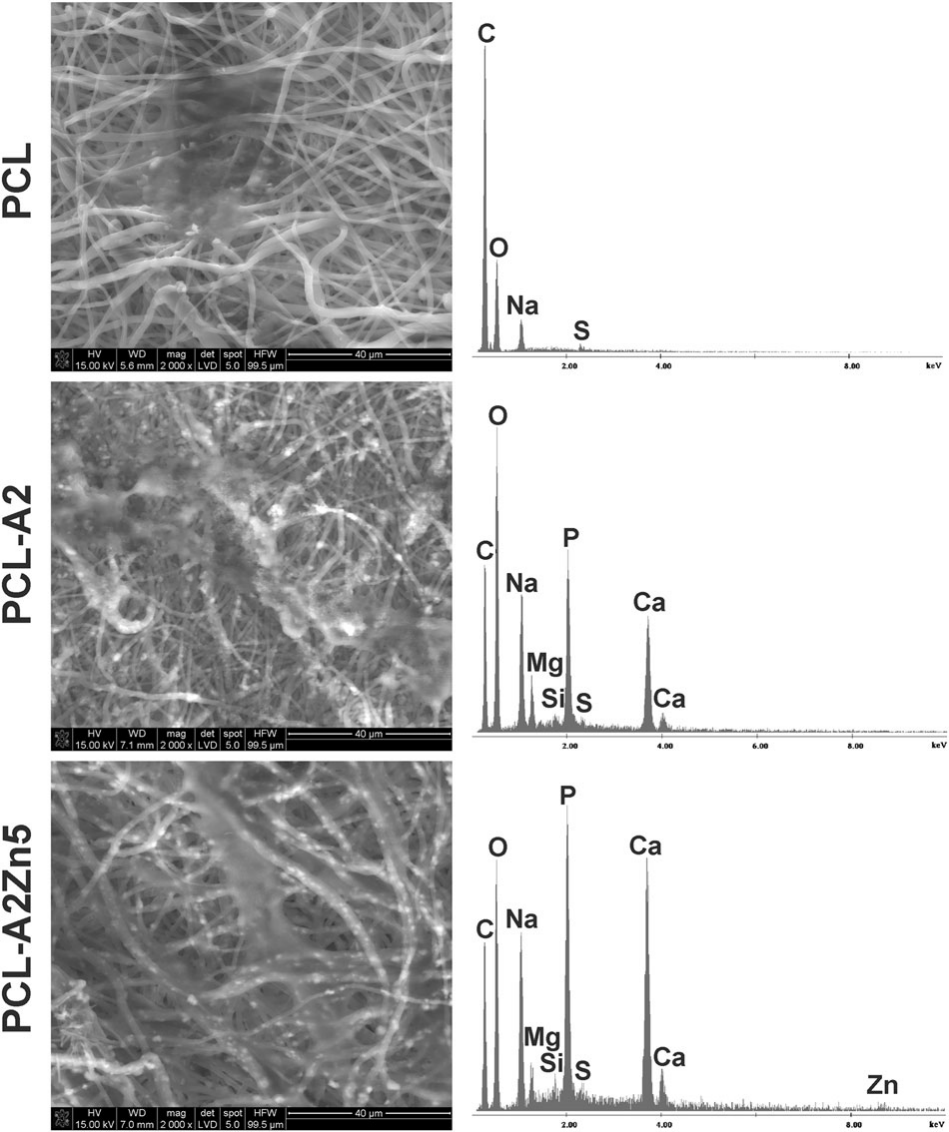
SEM images and EDX spectra of the PCL, PCL-A2 and PCL-A2Zn5 membranes with NHOst cells on the surface after 21 days of cells culture

Figure 6 show the SEM morphology of osteoblast cells on pure PCL and BG-modified membranes. Cells attached to the PCL-BG membranes revealed a normal spindle shape, similar to that of pure PCL membranes, indicating that the addition of Zn-doped BG did not affect cells’ morphology.

**Fig. 7:**
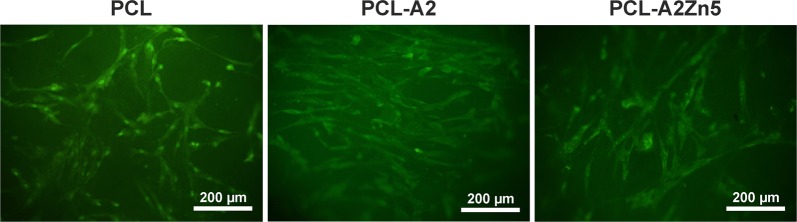
Fluorescence microscopy images of osteoblasts after 14 days of cells culture

Fluorescence microscopic images of NHOst cells adhered to membranes’ surface are shown in Fig. 7. NHOst cells spread well on the surface of membranes were evenly distributed and maintained their spindle morphology. The results indicated that the membranes possess good cytocompatibility and can support cells’ adhesion and spreading.

## Discussion

Particle size exerted significant effect on the morphology of the membranes. The smallest average diameter of the composite fibers (1.09 ± 0.5 µm) was obtained for the concentration of 4 wt% BG and a flow rate of 3 mL/h. The average fiber diameter was larger when Zn-doped BG was used. The reasons for this remain unclear. Although particles agglomerated, they were well distributed in the fibrous membrane. FTIR studies confirmed that A2 and A2Zn5 bioglass were successfully incorporated into the fibrous PCL membrane. This was supported by SEM images of the membranes containing BG.

As was shown in previous work [25], A2 gel-derived BG exhibits high surface reactivity in biologically related fluids. This is primarily due to a high calcium content which results in massive Ca^2+^ ion release from glass structure, leading to fast SBF supersaturation and thus high rate of hydroxyapatite nucleation and crystallization. Furthermore, it is well known that sol-gel-derived bioactive glasses exhibit high bioactivity because of their high surface area and the presence of siloxane groups (Si–OH) in their structure, providing favorable HAp nucleation sites [26, 27]. The results of bioactivity assessment demonstrated that the incorporation of BG particles into the PCL membrane plays an important role in the nucleation and growth of apatite layer on the surface of membranes. Some previous works revealed that the modification of bioactive glass with zinc retards HAp nucleation, especially at the early stage of incubation in SBF. This effect can result from absorption of Zn^2+^ ions (released from the glass structure) at the active growth sites of HAp, preventing its nucleation. The other explanation is that the solubility constant of Zn_3_(PO_4_)_2_ is lower (9.1 × 10^−33^) than the solubility constant of Ca_3_(PO_4_)_2_ (2 × 10^−29^), and therefore PO_4_^3−^ ions combine more easily with Zn^2+^ ions than with Ca^2+^ ions [28, 29]. Although Zn-doped bioactive glasses showed retarded apatite formation, Zn^2+^ ions released from their structure promote osteoblasts’ and chondrocytes’ differentiation, stimulate osteogenic differentiation of mesenchymal stem cells (MSCs) and bone matrix mineralization in vitro [15, 17, 30, 31], according to other authors.

Also, our results proved the influence of Zn^2+^ ions on the osteogenic differentiation of NHOst cells. However, comparing OI test values with the obtained values of cell number for the PCL-A2Zn5 sample, we conclude that the mineralization stage of the tested materials was achieved with a much smaller cell number. It means that the membranes which limited the proliferation capacity of cells also enhanced their differentiation and their ability to form hydroxyapatite. ALP activity also confirmed osteoblasts’ differentiation. The results indicate the potential of the tested materials in the initiation of cell differentiation and the cell mineralization processes. Zinc, when incorporated into apatite-based biomaterials, promoted attachment, growth and osteogenic differentiation of pre-osteoblasts [32] and various osteoblast-like cells [33–35].

Previous studies on hydrogel biomaterials (gellan gum, pectin) enhanced with A2 BG have demonstrated an antibacterial effect against methicilin-resistant Staphylococcus aureus (MRSA), one of the most commonly occuring antibiotic-resistant microbes in healthcare-related infections [36–38]. In one of the aforementioned studies, hydrogels containing A2Zn5 showed slightly greater antibacterial activity and equal cytocompatibility to hydrogels containing A2 BG [37]. Zn has also shown antibacterial activity as a component of calcium phosphate formed in gellan gum hydrogels [39]. What is more, Zn shows antibacterial and anti-inflammatory properties and inhibits osteoclasts differentiation [40]. Other authors have reported a positive influence of Zn on bone fracture healing [41]. The incorporation of zinc in various calcium phosphate-based biomaterials for bone regeneration led to higher new bone formation and bone–implant contact in vivo [42–45]. Other authors have reported a stimulatory effect of zinc on angiogenesis, which is desirable for bone healing [46].

## Conclusions

The BG and Zn-doped BG were successfully incorporated into the structure and onto the surface of fibrous membranes produced by electrospinning. The microstructure of PCL membranes were influenced by the bioglass size and parameters of the electrospinning process. It has been shown that 4% wt. of A2 BG concentration in the solution permits formation of membranes with uniform fiber diameter distribution. The SBF bioactivity test showed that the presence of A2 BG on the surface of electrospun membranes offered mineralization of the samples proving it bioactivity, however the presence of Zn ions was shown to induce a decrease in apatite precipitation. The mineralization and ALP activity were higher for polycaprolactone membranes modified with Zn-doped BG compared to pure PCL membranes or control material. Obtained results proved that the presence of Zn^2+^ ions in the electrospun membranes has a significant influence on the osteogenic differentiation of NHOst cells. PCL membranes modified with Zn doped bioglass in a layered nasal implant could act as a barrier membrane, which, coupled with a 3D printed bottom layer (for the cartilaginous part of implant), could allow for the proliferation of cells on both sides of the barrier, while at the same time preventing the unwanted migration of bone cells (from bony partition) across the barrier. Moreover, incorporation of zinc ions into nasal implants, could potentially provide antibacterial properties to decrease or eliminate bacterial adhesion on the implant surface.

## References

[1] Devaiah Anand K., Keojampa Bounmany Kyle (2009). Surgery of the Nasal Septum. Rhinology and Facial Plastic Surgery.

[2] Park SH, Yun BG, Won JY, Yun WS, Shim JH, Lim MH (2017). New application of three-dimensional printing biomaterial in nasal reconstruction. Laryngoscope.

[3] Takato T, Mori Y, Fujihara Y, Asawa Y, Nishizawa S, Kanazawa S (2014). Preclinical and clinical research on bone and cartilage regenerative medicine in oral and maxillofacial region. Oral Sci Int.

[4] Sykes JM, Patel KG (2008). Use of Medpor implants in rhinoplasty surgery. Oper Tech Otolaryngol – Head Neck Surg.

[5] Lin G, Lawson W (2007). Complication using grafts and implants in rhinoplasty. Oper Tech Otolaryngol – Head Neck Surg.

[6] Rajzer I, Kurowska A, Jabłoński A, Jatteau S, Śliwka M, Ziąbka M, Menaszek E (2018). Layered gelatin/PLLA scaffolds fabricated by electrospinning and 3D printing- for nasal cartilages and subchondral bone reconstruction. Mater Des.

[7] Ottoline ACX, Tomita S, Costa Marques MP, Felix F, Novaes Ferraiolo P, Santos Laurindo RS (2013). Antibiotic prophylaxis in otolaryngologic surgery. Int Arch Otorhinolaryngol.

[8] Park Young Jin, Cha Jong Hyun, Bang Sa Ik, Kim So Young (2018). Clinical Application of Three-Dimensionally Printed Biomaterial Polycaprolactone (PCL) in Augmentation Rhinoplasty. Aesthetic Plastic Surgery.

[9] Rajzer I, Menaszek E, Castano O (2017). Electrospun polymer scaffolds modified with drugs for tissue engineering. Mater Sci Eng C.

[10] Woodruff MA, Hutmacher DW (2010). The return of a forgotten polymer—polycaprolactone in the 21st century. Prog Polym Sci.

[11] Stoor P, Grénman R (2004). Bioactive glass and turbinate flaps in the repair of nasal septal perforations. Ann Oto Rhinol Laryngol.

[12] Vale AC, Pereira PR, Barbosa AM, Torrado E, Alves NM (2019). Optimization of silver-containing bioglass nanoparticles envisaging biomedical applications. Mater Sci Eng C.

[13] Zheng K, Boccaccini AR (2017). Sol-gel processing of bioactive glass nanoparticles: a review. Adv Colloid Interface Sci.

[14] Zheng K, Dai X, Lu M, Hüser N, Taccardi N, Boccaccini AR (2017). Synthesis of coppercontaining bioactive glass nanoparticles using a modified Stöber method for biomedical applications. Colloid Surface B.

[15] Su Yingchao, Cockerill Irsalan, Wang Yadong, Qin Yi-Xian, Chang Lingqian, Zheng Yufeng, Zhu Donghui (2019). Zinc-Based Biomaterials for Regeneration and Therapy. Trends in Biotechnology.

[16] Qiao Y, Zhang W, Tian P, Meng F, Zhu H, Jiang X, Liu X, Chu PK (2014). Stimulation of bone growth following zinc incorporation into biomaterials. Biomaterials.

[17] Wang T, Zhang JC, Chen Y, Xiao PG, Yang MS (2007). Effect of zinc ion on the osteogenic and adipogenic differentiation of mouse primary bone marrow stromal cells and the adipocytic trans-differentiation of mouse primary osteoblasts. J Trace Elem Med Biol.

[18] Bogun M, Mikolajczyk T, Kurzak A, Blazewicz M, Rajzer I. Influence of the As-spun draw ratio on the structure and properties of PAN fibres including montmorillonite. Fibres Text East Eur. 2006;14(2):13–6. http://www.fibtex.lodz.pl/article1171.html

[19] Rajzer I, Menaszek E, Bacakova L, Orzelski M, Błażewicz M (2013). Hyaluronic acid-coated carbon nonwoven fabrics as potential material for repair of osteochondral defects. Fibres Text East Eur.

[20] Rajzer I, Piekarczyk W, Castaño O (2016). An ultrasonic through-transmission technique for monitoring the setting of injectable calcium phosphate cement. Mater Sci Eng C.

[21] Domalik-Pyzik P, Morawska-Chochół A, Chłopek J, Rajzer I, Wrona A, Menaszek E, Ambroziak M (2016). Polylactide/polycaprolactone asymmetric membranes for guided bone regeneration. e-Polymers.

[22] Łączka M, Cholewa-Kowalska K, Kulgawczyk K, Klisch M, Mozgawa W (1999). Structural examinations of gel-derived materials of the CaO–P_2_O_5_–SiO_2_ system. J Mol Struct.

[23] Dziadek M, Zagrajczuk B, Menaszek E, Wegrzynowicz A, Pawlik J, Cholewa-Kowalska K (2016). Gel-derived SiO_2_–CaO–P_2_O_5_ bioactive glasses and glass-ceramics modified by SrO addition. Ceram Int.

[24] Kokubo T, Takadama H (2006). How useful is SBF in predicting in vivo bone bioactivity?. Biomaterials.

[25] Dziadek M, Zagrajczuk B, Menaszek E, Cholewa-Kowalska K (2018). A new insight into in vitro behaviour of poly(ε-caprolactone)/bioactive glass composites in biologically related fluids. J Mater Sci.

[26] Mami M, Lucas-Girot A, Oudadesse H, Dorbez-Sridi R, Mezahi F, Dietrich E (2008). Investigation of the surface reactivity of a sol–gel derived glass in the ternary system SiO_2_–CaO–P_2_O_5_. Appl Surf Sci.

[27] Dziadek M, Zagrajczuk B, Menaszek E, Dziadek K, Cholewa-Kowalska K (2017). Poly(ε-caprolactone)-based membranes with tunable physicochemical, bioactive and osteoinductive properties. J Mater Sci.

[28] Balasubramanian P, Strobel LA, Kneser U, Boccaccini AR (2015). (2015) Zinc-containing bioactive glasses for bone, dental and orthopedic applications. Biomed Glasses.

[29] Du RL, Chang J, Ni SY, Zhai WY, Wang JY (2006). Characterization and in vitro bioactivity of zinc-containing bioactive glass and glass-ceramics. J Biomater Appl.

[30] Oh SA, Kim SH, Won JE, Kim JJ, Shin US, Kim HW (2010). Effects on growth and osteogenic differentiation of mesenchymal stem cells by the zinc-added sol-gel bioactive glass granules. J Tissue Eng.

[31] Wang X, Li X, Ito A, Sogo Y (2011). Synthesis and characterization of hierarchically macroporous and mesoporous CaO–MO–SiO_2_–P_2_O (M = Mg, Zn, Sr) bioactive glass scaffolds. Acta Biomater.

[32] Storrie H, Stupp SI (2005). Cellular response to zinc-containing organoapatite: an in vitro study of proliferation, alkaline phosphatase activity and biomineralization. Biomaterials.

[33] Webster TJ, Ergun C, Doremus RH, Bizios R (2002). Hydroxylapatite with substituted magnesium, zinc, cadmium, and yttrium. II. Mechanisms of osteoblast adhesion. J Biomed Mater Res.

[34] Yang F, Dong WJ, He FM, Wang XX, Zhao SF, Yang GL (2012). Osteoblast response to porous titanium surfaces coated with zinc-substituted hydroxyapatite. Oral Surg Oral Med Oral Pathol Oral Radiol.

[35] Wang X, Ito A, Sogo Y, Li X, Oyane A (2010). Zinc-containing apatite layers on external fixation rods promoting cell activity. Acta Biomater.

[36] Douglas TE, Piwowarczyk W, Pamula E, Liskova J, Schaubroeck D, Leeuwenburgh SC (2014). Injectable self-gelling composites for bone tissue engineering based on gellan gum hydrogel enriched with different bioglasses. Biomed Mater.

[37] Douglas TEL, Dziadek M, Gorodzha S, Lišková J, Brackman G, Vanhoorne V (2018). Novel injectable gellan gum hydrogel composites incorporating Zn- and Sr-enriched bioactive glass microparticles: high-resolution X-ray microcomputed tomography, antibacterial and in vitro testing. J Tissue Eng Regen Med.

[38] Douglas TEL, Dziadek M, Schietse J, Boone M, Declercq HA, Coenye T (2019). Pectin-bioactive glass self-gelling, injectable composites with high antibacterial activity. Carbohydr Polym.

[39] Douglas TEL, Pilarz M, Lopez‐Heredia M, Brackman G, Schaubroeck D, Balcaen L (2017). Composites of gellan gum hydrogel enzymatically mineralized with calcium-zinc phosphate for bone regeneration with antibacterial activity. J Tissue Eng Regen Med.

[40] Bejarano J, Caviedes P, Palza H (2015). Sol-gel synthesis and in vitro bioactivity of copper and zinc-doped silicate bioactive glasses and glass-ceramics. Biomed Mater.

[41] Krell ES, Ippolito JA, Montemurro NJ, Lim PH, Vincent RA, Hrehaet J (2017). Local zinc chloride release from a calcium sulfate carrier enhances fracture healing. J Orthop Trauma.

[42] Kawamura H, Ito A, Miyakawa S, Layrolle P, Ojima K, Ichinose N, Tateishi T (2000). Stimulatory effect of zinc-releasing calcium phosphate implant on bone formation in rabbit femora. J Biomed Mater Res.

[43] Kawamura H, Ito A, Muramatsu T, Miyakawa S, Ochiai N, Tateishi T (2003). Long-term implantation of zinc-releasing calcium phosphate ceramics in rabbit femora. J Biomed Mater Res A.

[44] Li X, Sogo Y, Ito A, Mutsuzaki H, Ochiai N, Kobayashi T, Nakamura S, Yamashita K, Legeros RZ (2009). The optimum zinc content in set calcium phosphate cement for promoting bone formation in vivo. Mater Sci Eng C.

[45] Pina S, Vieira SI, Rego P, Torres PMC, da Cruz e Silva OAB, da Cruz e Silva EF, Ferreira JMF (2010). Biological responses of brushite-forming Zn- and ZnSr- substituted beta-tricalcium phosphate bone cements. Eur Cell Mater.

[46] Imai K, Nishikawa T, Tanaka A, Suese K, Takashima H, Takeda S (2010). In vitro new capillary formation with eight metal ions of dental biomaterials. J Oral Tissue Eng.

